# The involvement of CiaR and the CiaR-regulated serine protease HtrA in thermal adaptation of *Streptococcus pneumoniae*

**DOI:** 10.1099/mic.0.001304

**Published:** 2023-02-22

**Authors:** Ozcan Gazioglu, Medhanie Habtom, Peter W. Andrew, Hasan Yesilkaya

**Affiliations:** ^1^​ Department of Respiratory Sciences, University of Leicester, Leicester, UK

**Keywords:** CiaRH, HtrA, *Streptococcus pneumoniae*, thermal regulation, virulence

## Abstract

The *in vivo* temperature can vary according to the host tissue and the response to infection. *Streptococcus pneumoniae* has evolved mechanisms to survive these temperature differences, but neither the consequences of different temperatures for pneumococcal phenotype nor the genetic basis of thermal adaptation are known in detail. In our previous study [16], we found that CiaR, which is a part of two-component regulatory system CiaRH, as well as 17 genes known to be controlled by CiaRH, were identified to be differentially expressed with temperature. One of the CiaRH-regulated genes shown to be differentially regulated by temperature is for the high-temperature requirement protein (HtrA), coded by SPD_2068 (*htrA*). In this study, we hypothesized that the CiaRH system plays an important role in pneumococcal thermal adaptation through its control over *htrA*. This hypothesis was evaluated by testing strains mutated or overexpressing *ciaR* and/or *htrA*, in *in vitro* and *in vivo* assays. The results showed that in the absence of *ciaR*, the growth, haemolytic activity, amount of capsule and biofilm formation were considerably diminished at 40 °C only, while the cell size and virulence were affected at both 34 and 40 °C. The overexpression of *htrA* in the ∆*ciaR* background reconstituted the growth at all temperatures, and the haemolytic activity, biofilm formation and virulence of ∆*ciaR* partially at 40 °C. We also showed that overexpression of *htrA* in the wild-type promoted pneumococcal virulence at 40 °C, while the increase of capsule was observed at 34 °C, suggesting that the role of *htrA* changes at different temperatures. Our data suggest that CiaR and HtrA play an important role in pneumococcal thermal adaptation.

## Introduction

*Streptococcus pneumoniae* is a frequent asymptomatic colonizer of the human upper respiratory tract. The microbe can disseminate from the upper respiratory tract to other niches (lung, ear, blood, brain), which can lead to serious diseases, including otitis media, pneumonia, bacteraemia, meningitis, or adverse cardiac events [1]. During dissemination, the pneumococci are challenged by many environmental stresses, such as nutrient deprivation, variation in concentration of oxygen or pH and host stress hormones [2], as well as a range of temperatures [3]. *In vivo* survival is dependent on pneumococcal adaptive responses to fluctuating host environments, but our knowledge of these traits is limited.

In different host tissues, the pneumococcus is exposed to different temperatures. The average temperature increases from 30–34 °C through the anterior to the posterior section of the nasopharynx [3, 4]. The core body temperature is 37 °C, and it increases up to 39–40 °C during pneumococcal infection or viral co-infection, due to an inflammatory response [5]. The pneumococcus senses and responds to fluctuations in temperature by the synthesis of proteins known as heat shock proteins (HSPs) and RNA thermosensors [6, 7]. HSPs act as chaperones or proteases, and are involved in controlling the quality of newly synthesized proteins, refolding denatured or unfolded proteins, and preventing the accumulation of damaged proteins under temperature stress [8]. Some chaperones, such as GroEL, DnaK and Clp, have elevated expression when exposed to environmental stress in the pneumococcus [8], similar to many other bacterial species, for example *Mycobacterium smegmatis* [9], *Campylobacter jejuni* [10] and *Salmonella enterica* serovar Typhimurium [11]. RNA is considered to be a thermosensor due to its changeable physical properties at different temperatures [12]. RNA thermosensors (RNATs) are found in the 5′ untranslated region (UTR) of bacterial heat and cold shock genes, and virulence genes [6]. Recently, two novel RNATs, linked to CpsA, which is responsible for the synthesis of pneumococcal capsular polysaccharide, and PspC encoding the production of factor H protein, have been identified [12]. These RNATs enable microbe escape from temperature-dependent immune evasion.

In our previous study [13], we characterized the pneumococcal *gdhA,* which encodes glutamate dehydrogenase, as it was indicated that glutamate dehydrogenase is a thermostable protein in hyperthermophiles [14] and has an additional role in other stress conditions [15]. We found that the *gdhA* is temperature-responsive and is required for pneumococcal metabolism and virulence at 40 °C [13]. Our transcriptomic data demonstrated that 252 pneumococcal genes were expressed differently when exposed to 34 and 40 °C, relative to 37 °C at mid-exponential phase of growth: 97 genes were upregulated, while 35 genes were downregulated at 34 °C, affecting the expression of 25 operons. Further analysis of the pneumococcal transcriptome revealed that most of the differentially expressed genes were encoding proteins involved in competence, purine/pyrimidine metabolism, bacteriocin synthesis and transcriptional regulation, and hypothetical proteins with unknown functions [13]. One of the genes differentially expressed at different temperatures was CiaR (SPD_0701), which is the response regulator of CiaRH two-component regulatory system (TCS) in *S. pneumoniae *. It was found to be upregulated at 34 °C (2.1-fold) but downregulated at 40 °C (2.6-fold), relative to 37 °C. CiaRH (TCS05) is highly conserved in the streptococcal genome [16] and has been studied in various streptococci, including *S. pneumoniae *. It has been shown that CiaRH is involved in competence development, biofilm formation, antibiotic resistance, autolysis, cell wall biosynthesis, bacteriocin production, tolerance to oxidative and acid stress, and virulence [17–21].

In the pneumococcus, CiaRH plays a role on biofilm formation *in vivo* [22], controls the transcription of *nanA* and the sialic acid transporter genes *sat*ABC, mediates pneumococcal colonization in the presence of N-acetylneuraminic acid, and contributes to virulence [23] and the oxidative stress response [18]. As shown in Table S1(a) (available in the online version of this article), the CiaRH regulon controls 18 operons in *S. pneumoniae* [21, 24, 25], as revealed by using motif analysis of repeat sequence (TTTAAG-N5-TTTAAG) in the promoter regions of regulated genes [26]. In addition to differential expression of CiaRH, we identified 17 genes that are controlled by CiaRH at 34 and 40 °C, relative to 37 °C (Table S1b) [13]. These genes putatively code for bacteriocin production, competence development, stress response and hypothetical proteins with unknown functions. While three genes, SPD_0775, SPD_1131 and SPD_2006, were only upregulated at 34 °C, the rest were upregulated at 34 °C (3.2 to 53.6-fold) and downregulated at 40 °C (2.7 to 44.9-fold) relative to 37 °C.

In this study, we hypothesized that CiaRH is involved in pneumococcal thermal adaptation that occurs through CiaRH’s control over *htrA* encoding serine protease. The reason we focused on HtrA-like serine protease was that it was shown that HtrA plays an important role in stress response, including oxidative and antimicrobial stresses [18, 27–29]. This serine protease is upregulated at 34 °C (6.4-fold) and downregulated at 40 °C (3.7-fold) [13]. Although HtrA is known as a heat shock serine protease that participates in degradation of misfolded proteins at high-temperature stress, it was reported that the HtrA homologue DegP in *Escherichia coli* acts as a protease and a chaperone to protect cells at both low and high temperatures [30].

Here, we studied the role of CiaRH and HtrA in pneumococcal thermal adaptation by testing the phenotypic traits shown to be influenced by temperature, such as growth, cell size, biofilm formation, capsule synthesis, pneumolysin activity and virulence, in *Galleria mellonella* [13]. We found that at the high and low temperatures tested, CiaRH contributes to pneumococcal thermal adaptation, and this contribution is mediated partly through the effector action of HtrA.

## Methods

### Bacterial strains and growth conditions

Bacterial strains and plasmids used in this study are listed in Table S2. Pneumococci were grown either micro-aerobically in brain heart infusion (BHI) broth, on blood agar plates supplemented with 5 % (v/v) defibrinated horse blood, Todd–Hewitt broth (THB) or in THB supplemented with 0.5 % (w/v) yeast extract (THY). The cultures were incubated at 34 °C, 37 °C, or 40 °C, as appropriate. In addition, chemically defined medium (CDM) supplemented with 55 mM glucose was also used for characterization of pneumococcal strains [31]. Where appropriate, spectinomycin (100 µg ml^−1^) or kanamycin (50 µg ml^−1^) was added to the culture medium for selection. *E. coli* strain Top10 (Invitrogen) was used for cloning and was grown in lysogeny broth (LB) at 37 °C in a shaking incubator at 200 r.p.m. or on LB agar, supplemented with kanamycin (50 µg ml^−1^) or ampicillin (100 µg ml^−1^).

### Construction of genetically modified strains

A *ciaR* (SPD_0701) deletion mutant was constructed using the splicing by overlap extension (SOEing) PCR method and the primers listed in Table S3 [32]. Briefly, up- and downstream flanking regions of the target gene were amplified and fused with a spectinomycin resistance gene amplified from pDL278 [33]. The fused fragments were then transformed into the *S. pneumoniae* D39 genome and the mutant strain was designated *∆ciaR*. To rule out any polar effect of mutagenesis, an intact copy of the *ciaR* coding sequence was introduced into the pneumococcal genome at a transcriptionally silent site using the non-replicative pneumococci plasmid pCEP as described previously [31]. In addition, to study the impact of CiaR-regulated *htrA* on thermal adaptation of the pneumococcus, the native promoter and coding sequence of *htrA* was introduced into *∆ciaR* (*htrA:: ∆ciaRcomp*) and D39 wild-type strain (*htrA::htrA-*wt).

### Haemolytic activity assay

The haemolytic activity of pneumococcal strains was obtained at different temperatures, as described in our previous publication [13]. Pneumococcal cell lysates were prepared by sonication at an amplitude of 8 µm for 15 s on and 45 s off (Soniprep 150). Fifty microlitres of serially diluted lysates in phosphate-buffered saline (PBS) (pH 7.0) were mixed with 4 % (v/v) sheep red blood cells (RBCs) (Oxoid) and incubated at 37 °C for 30 min, and haemolysis was observed by eye. The haemolytic units (HU) were calculated as the highest dilution of lysate causing 50 % lysis of RBCs using the standard curve and normalized against total protein concentration.

### Capsular polysaccharide (CPS) quantification

The impact of different temperatures on pneumococcal capsule was quantified by the production of glucuronic acid [31]. The pneumococcal strains were grown to mid-exponential growth phase (OD_600_ 0.5–0.7) in CDM supplemented with glucose and mixed with 100 µl of 1 % (v/v) Zwittergent 3–14 detergent (Sigma-Aldrich) in 100 mM citric acid (pH 2.0). The mixture was then incubated at 50 °C for 20 min and the polysaccharides were precipitated in 1 ml of absolute ethanol. The pellet was dissolved in 200 µl of distilled water and mixed with 1.2 ml of 12.5 mM borax (Sigma-Aldrich) in H_2_SO_4_. The mixture was boiled at 100 °C for 5 min, cooled to room temperature and mixed with 20 µl of 0.15 % (w/v) 3-hydroxydiphenol (Sigma-Aldrich). Absorbance was recorded at 520 nm and the amount of glucuronic acid was calculated by comparison to a standard curve generated with known concentrations of glucuronic acid and normalized against per 10^9^ c.f.u.

### Biofilm formation assay

Biofilm formation by pneumococcal strains was analysed using the crystal violet attachment assay [34]. A pellet of overnight pneumococcal culture grown in THY medium in a 12-well plate was resuspended in 2 ml fresh THY. The culture was then serially diluted to obtain an OD_600_ of 0.05–0.1. After overnight growth, the excess medium was carefully aspirated, and biofilms were washed gently with 200 µl PBS three times to remove weakly or non-adherent bacteria. Attached cells were stained with 50 µl of 0.1 % (w/v) crystal violet for 15 min, excess stain was discarded, and the biofilms were washed with distilled water three times. Subsequently, biofilm was dissolved in 200 µl of 95 % (v/v) ethanol and the absorbance was measured at 595 nm. The amount of biofilm formed was expressed as absorbance per 10^8^ c.f.u.

### Determination of cell size

Pneumococcal strains were grown overnight in CDM supplemented with 55 mM glucose. Bacterial suspensions were Gram-stained as previously described [34] . Pneumococcal cell size was measured length-wise using a Prior microscope equipped with a digital camera (Infinity) and image analysis software (Infinity). For each assay at least 50 cells were analysed.

### *G. mellonella* model of pneumococcal infection

Larvae were acquired from Livefood, UK, and those weighing 25–30 mg with a white, milky appearance were used for infection. First, 5×10^5^ c.f.u. pneumococci prepared in 10 µl of PBS were administered to the second pro-leg of the larvae. For each strain at each temperature, 10 larvae were injected. In addition, a control group of larvae was injected with 10 µl of PBS. Infected larvae were incubated at the respective growth temperature and the mortality numbers were recorded at 24 h post-infection.

### Statistical analysis

GraphPad prism version 8 (GraphPad, CA, USA) was used for data analysis. All experiments were performed in triplicate on at least three separate occasions and the results were described as mean±standard error of the mean (sem). Where appropriate, one- or two-way analysis of variance (ANOVA) followed by Tukey’s multiple comparison test was used to compare the groups. A *P* value <0.05 was taken as showing significance.

## Results

### Overexpression of *htrA* restores the attenuated growth of ∆*ciaR* at high temperature

**Fig. 1.**
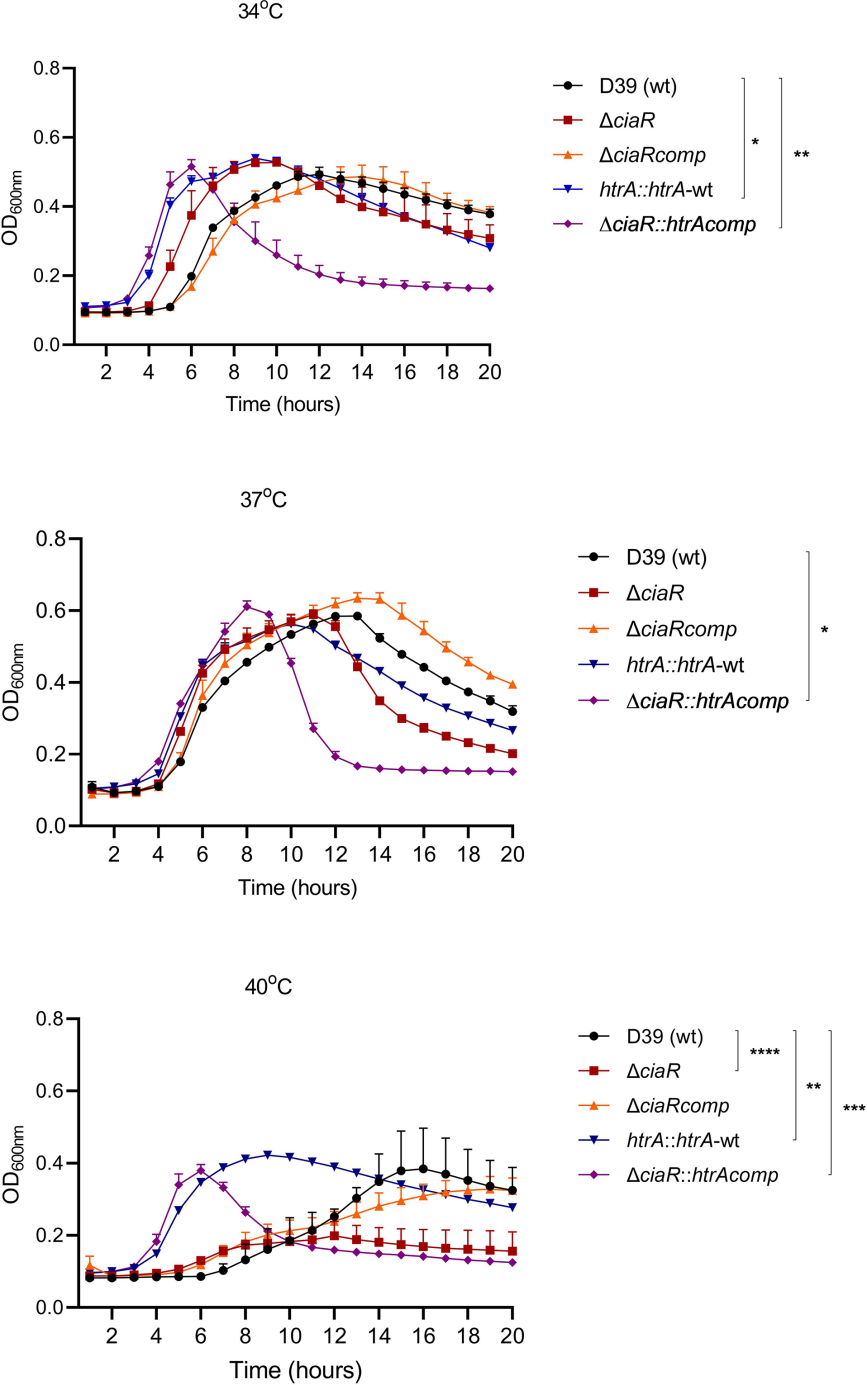
Growth profiles of pneumococcal strains in CDM supplemented with 55 mM glucose at 34, 37, or 40 °C. Error bars show the standard error of the mean for three individual replicates of nine independent biological samples. Significant differences were seen between the growth rate of the wild-type D39 and other pneumococcal strains at different temperatures using ANOVA followed by Tukey’s multiple comparison test (*P<0.05, **P<0.01, ***P<0.001, ****P<0.0001).

To determine the involvement of CiaR and HtrA in growth at different temperatures, pneumococcal strains were grown in CDM supplemented with 55 mM glucose at 34, 37 and 40 °C (Fig. 1). We found that the growth rate of the wild-type was similar at 34 and 37°C but reduced at 40 °C compared to 37 °C (*P*<0.05). The growth of ∆*ciaR* was significantly diminished at 40 °C compared to 34 and 37 °C, with a lower growth rate and yield than the wild-type (*P*<0.05). The complemented strain, ∆*ciaRcomp*, had a similar growth profile to the wild-type at all temperatures. The ∆*ciaR*-overexpressing *htrA* strain, ∆*ciaR::htrAcomp*, had a similar growth rate at all temperatures, but the yield was significantly less at 40 °C compared to other temperatures (*P*<0.05). The growth rate of ∆*ciaR::htrAcomp* was significantly higher compared to ∆*ciaR* and the wild-type at all temperatures (*P*<0.01 and *P*<0.001, respectively). ∆*ciaR::htrAcomp* also had a reduced lag phase at 40 °C, and autolysed earlier than the wild-type at all temperatures. Overexpression of *htrA* in the wild-type background, *htrA::htrA*-wt, led to a similar growth rate at 34 and 37 °C, but a lower rate at 40 °C (*P*<0.01). Compared to the wild-type, *htrA::htrA*-wt had a shorter lag phase and a significantly higher growth rate at 34 and 40 °C (*P*<0.05).

These data show that the growth of the wild-type is affected at 40 °C, and *ciaR* is required for growth at this temperature. Overexpression of *htrA* in wild-type and ∆*ciaR* strains promotes growth, particularly at 40 °C compared to 34 °C, suggesting that CiaR-mediated thermal adaptation occurs through the action of *htrA*.

After establishing a role for CiaR in pneumococcal growth at different temperatures, we decided to test the contribution of CiaR on different phenotypes that are known to be influenced by temperature: cell size, biofilm formation, haemolytic activity, capsule biosynthesis and virulence [13].

### CiaR, but not HtrA, is required to adjust the cell size at different temperatures

The pneumococcal strains were grown in glucose at different temperatures and cell size was measured. The temperature shifts led to a statistically significant decrease in the cell size of the wild-type at 40 °C (0.23±0.03 µm) compared to 34 and 37 °C (0.29±0.03 and 0.32±0.03 µm, respectively, *n*=50) (*P*<0.01). When *ciaR* was deleted, the cell size decreased at 34 °C (0.21±0.01 µm *n*=50) and 40 °C (0.17±0.02 µm) relative to 37 °C (0.28±0.03 µm *n*=50), but the reduction was more pronounced at 40 °C against 37 °C. In comparison, ∆*ciaR* cells were significantly smaller than the wild-type at 34 and 40 °C (*P*<0.05). Moreover, no difference was observed between the wild-type and the complemented mutant strain (∆*ciaRcomp*) at any growth temperature (for 34, 37 and 40 °C: 0.29±0.02, 0.32±0.02 and 0.24±0.01 µm, respectively, *P*>0.05, at each temperature). The overexpression of *htrA* in the wild-type did not have any significant effect on cell size, regardless of growth temperature (*P*>0.05). Similarly, in ∆*ciaR*, *htrA* overexpression did not reconstitute the cell size at any temperature (at 34 °C: 0.22±0.02 µm, 37 °C: 0.27±0.01 µm, 40 °C: 0.18±0.01 µm, *n*=50). In conclusion, CiaR is required at different temperatures to adjust the cell size of pneumococcus and HtrA has no significant role in the cell size.

### CiaR and HtrA are involved in pneumococcal biofilm formation at high temperature

**Fig. 2.**
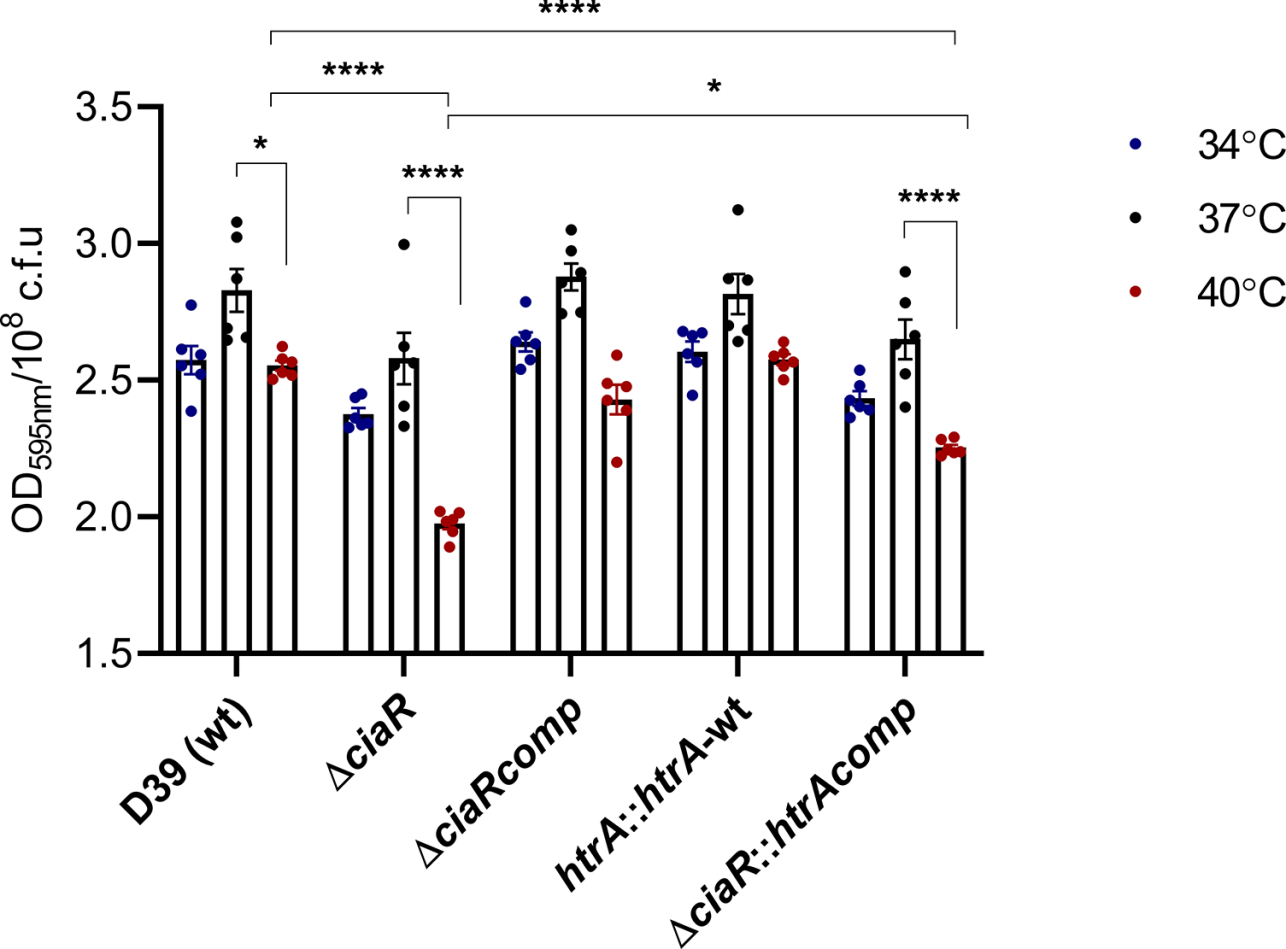
The biofilm formation of pneumococcal strains tested at different temperatures using crystal violet assay on overnight cultures grown in static conditions. All values are expressed as the optical density of stained adherent cells at 595 nm. Each column represents the means of three individual measurements, each with triplicates, with their standard error of means. Mean differences in biofilm formation of the mutant strain were compared to the wild-type strain and amongst themselves at tested temperatures using ANOVA and Tukey’s multiple comparisons tests (*P<0.05, ****P<0.0001).

The average temperature of the nasopharynx in the human host is 34 °C and pneumococci reside in the nasopharynx in well-organized biofilms [35]. In this study, we measured the biofilm formation of pneumococcal strains at different temperatures (Fig. 2). The wild-type, ∆*ciaR* and *∆ciaR::htrAcomp* strains produced significantly less biofilm at 40 °C compared to at 34 and 37 °C (*P*<0.05). Overexpressing *htrA* in the wild-type did not affect the amount of biofilm at any temperature (*P*>0.05), but *htrA* overexpression in the ∆*ciaR* increased the biofilm formation at 40 °C (*P*<0.05). These results show that the biofilm formation was disrupted at elevated temperature and *ciaR* is required for forming biofilm under temperature stress. In addition, *htrA* is able to promote biofilm formation in ∆*ciaR* at 40 °C.

### The haemolytic activity is controlled by CiaR at 40 °C

**Fig. 3.**
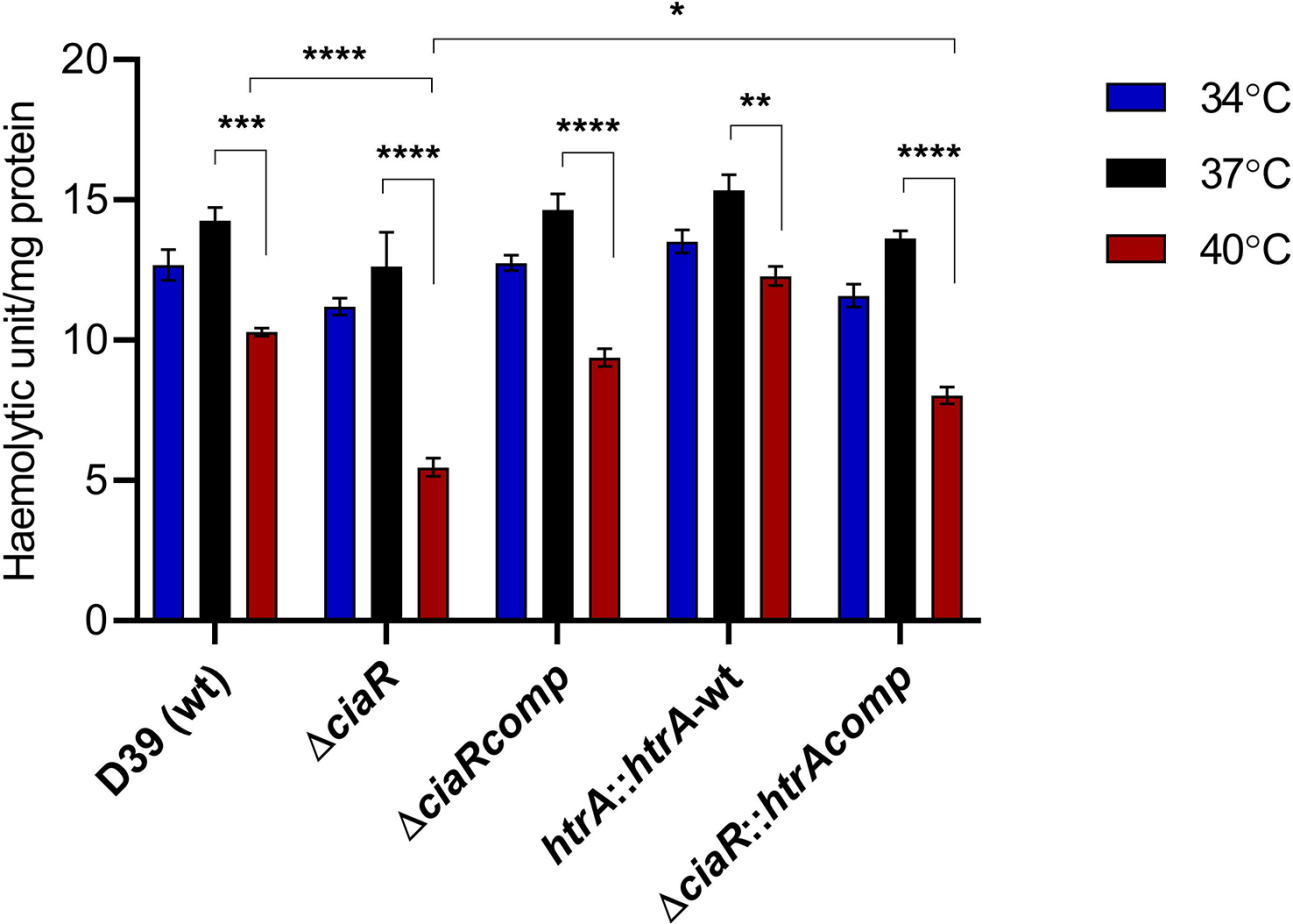
The haemolytic activity of pneumococcal strains grown in CDM supplemented with 55 mM glucose at different temperatures. Pneumolysin activity was measured observing haemolytic activity on 4 % v/v defibrinated sheep blood. Error bars show the standard error of the mean for three individual measurements, each with three independent biological samples. Significance at different temperatures was calculated using ANOVA and Tukey’s multiple comparisons tests. (*P<0.05, **P<0.01, ***P<0.001, ****P<0.0001).

Previously it was shown that virulence gene expression is influenced by the growth temperature of *S. pneumoniae* [5]. Therefore, we tested the production of two well-known pneumococcal virulence factors, pneumolysin and capsule, in pneumococcal strains at different temperatures. In this study, pneumolysin was measured by the haemolysis of RBCs using pneumococcal cell lysates harvested from the cultures grown in CDM supplemented with 55 mM glucose until mid-exponential growth phase at different temperatures. The results showed that all the pneumococcal strains had lower haemolytic activity at 40 °C relative to their activity at 34 and 37 °C (*P*<0.05), while no difference was recorded between 34 and 37 °C (*P*>0.05) (Fig. 3). In the absence of *ciaR*, haemolysis was significantly diminished compared to the wild-type at 40 °C (*P*<0.0001). Overexpressing *htrA* in D39 did not significantly increase the level of haemolysis at any temperature, relative to the wild-type. Moreover, an additional copy of *htrA* in ∆*ciaR* restored the activity at 40 °C. These results suggest that CiaR is needed for pneumolysin activity at higher temperature, probably through its control over *htrA*.

### CiaR controls capsule synthesis at different temperatures and HtrA plays a controlling role in determining capsule level at the lower temperature

**Fig. 4.**
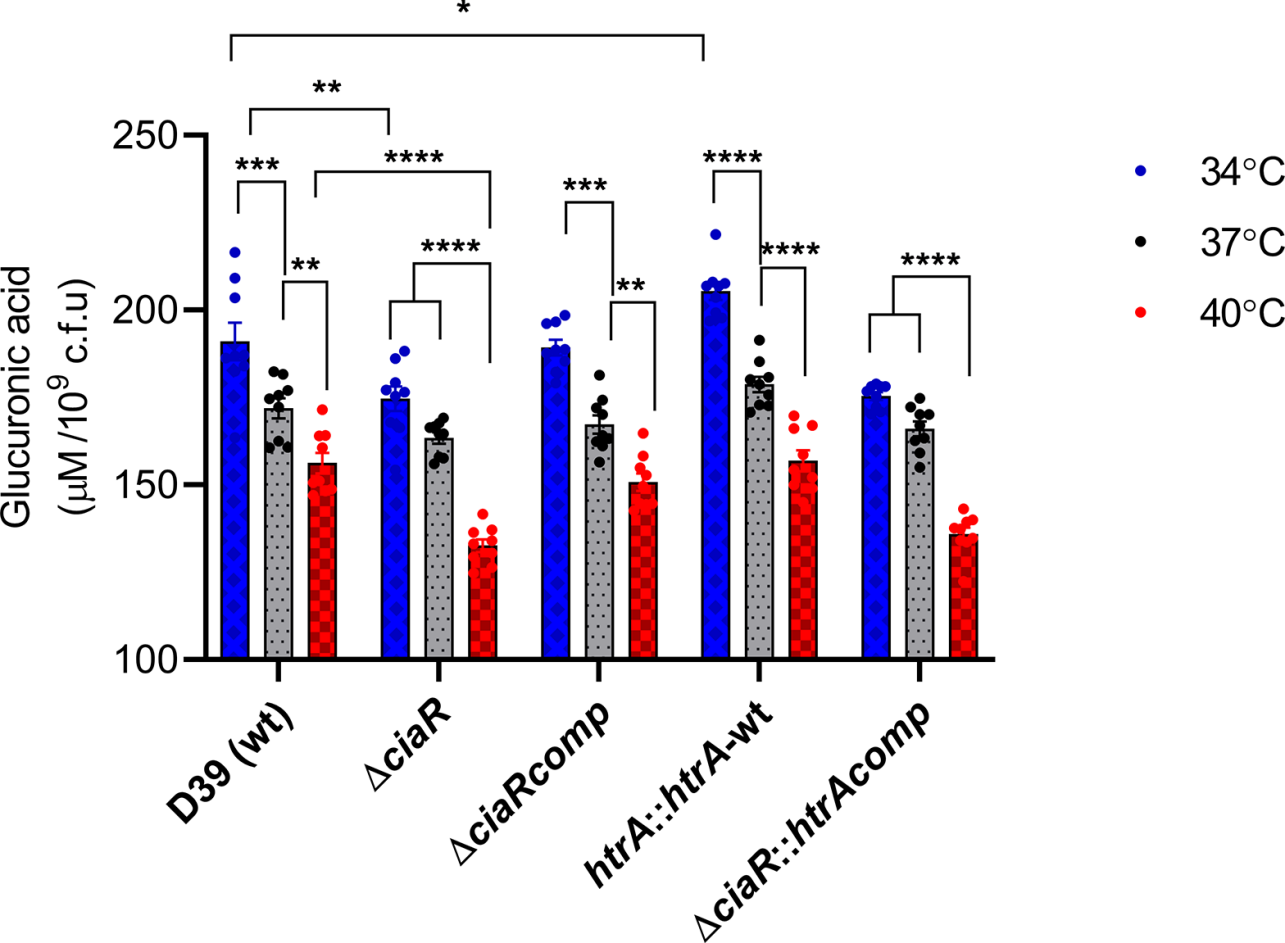
Glucuronic acid concentration of pneumococcal strains grown in CDM supplemented with 55 mM glucose was quantified to compare the amount of capsule at different temperatures. Significant differences were seen using ANOVA and Tukey’s multiple comparisons tests. Error bars show the standard error of the mean for three individual measurements, each with three independent experiments (*P<0.05, **P<0.01, ***P<0.001, ****P<0.0001).

Capsular polysaccharide (CPS) is a major pneumococcal virulence determinant that has multi-functional roles in different host niches [1]. Glucuronic acid is a component of type 2 capsule and its quantity was assayed to determine capsule production at different temperatures. The results in Fig. 4 showed that when the wild-type strain was grown in CDM, it produced a larger amount of glucuronic acid at 34 °C than 37 and 40 °C (*P*<0.05) The ∆*ciaR* strain produced a similar amount of glucuronic acid at 34 and 37 °C, but significantly higher amounts at 40 °C (*P*<0.0001). Compared to the wild-type, ∆*ciaR* produced less capsule at 34 °C (*P*<0.01) and 40 °C (*P*<0.0001), but not at 37 °C (*P*>0.05). In addition, the complemented ∆*ciaR* showed a similar phenotype to the wild-type. When *htrA* was overexpressed in D39, the amount of capsule was significantly higher at 34 °C than at 37 and 40 °C (*P*<0.0001). Moreover, at 34 °C, the *htrA::htrA-*wt had a higher amount of capsule compared to the wild-type (*P*<0.05). The *∆ciaR::htrAcomp* strain produced a similar level of glucuronic acid at 34 and 37 °C compared to 40 °C (*P*<0.0001). There was no difference in the glucuronic acid content of ∆*ciaR* and *∆ciaR::htrAcomp* at any temperature.

The results show that the production of pneumococcal capsule is affected by the temperature and CiaR is required for capsule synthesis at 34 and 40 °C. The presence of an additional copy of *htrA* increased the level of glucuronic acid in the wild-type background at 34 °C, suggesting that *htrA* also has an impact on the capsule synthesis at the lower temperature in *S. pneumoniae *. However, *htrA* overexpression did not have any effect on capsule synthesis in the *∆ciaR* background at any temperature, suggesting that CiaR control over capsule is independent of HtrA.

### Pneumococcal virulence is controlled by CiaR through overexpression of *htra* at high temperature

**Fig. 5.**
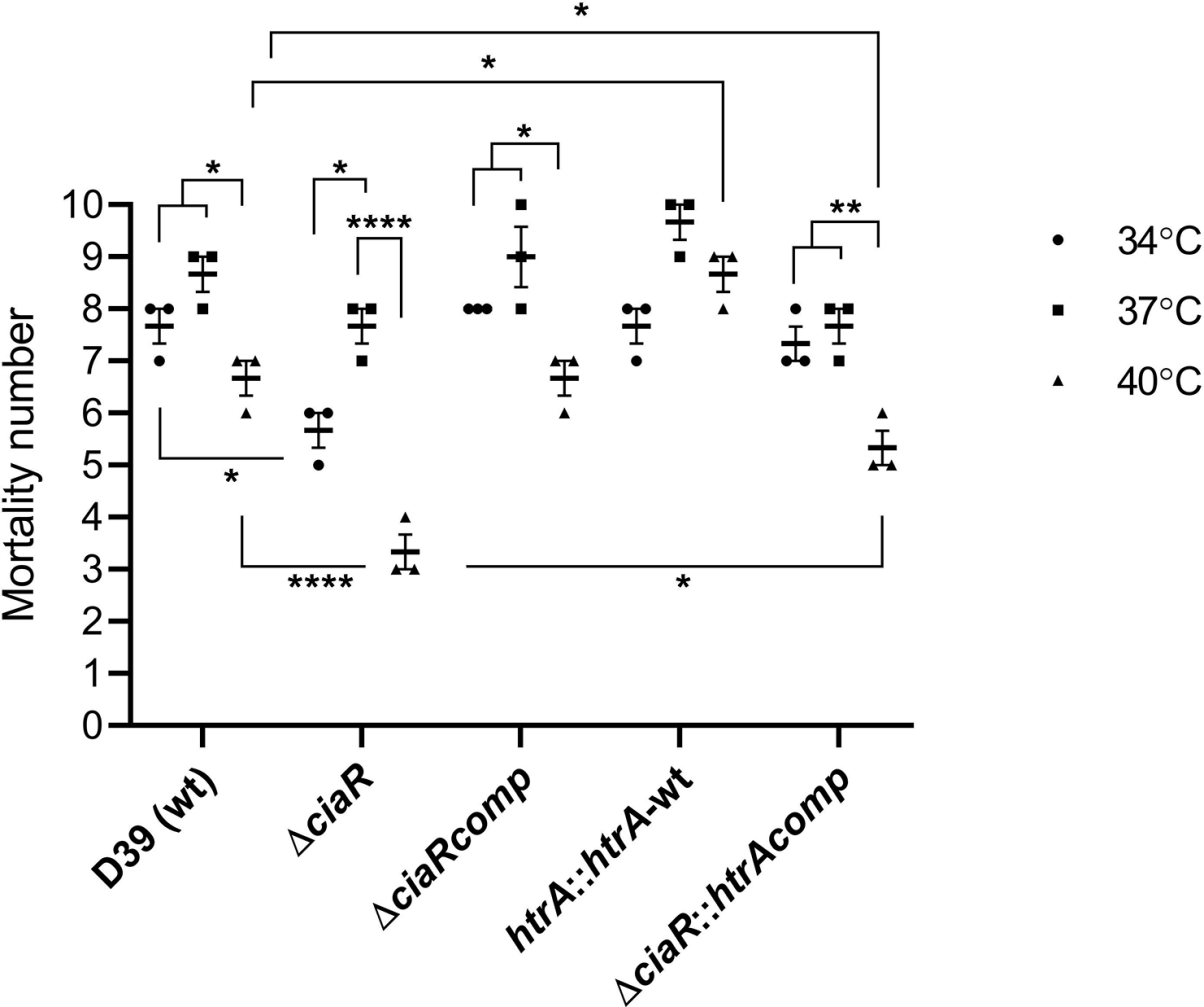
*In vivo* test of pneumococcal strains at different temperatures defining survival of *G. mellonella* infected with 5×10^5^ c.f.u./larva. Each dot represents the number of dead larvae for individual group (*n*=10) at 34 (circle), 37 (square), or 40 °C (triangle). Significant differences in mortality numbers are seen comparing the D39 wild-type strain with the mutant and complemented strains using ANOVA and Tukey’s multiple comparisons tests. Error bars show the standard error of the mean. (*P<0.05, **P<0.01, ****P<0.0001).

*In vitro* analysis of pneumococcal strains showed that temperature can have an impact on growth, cell size, production of virulence determinants and biofilm formation. To investigate a potential role of *ciaR* and *htrA in vivo*, we used *G. mellonella* as an infection model. The advantage of using waxworms is their ability to survive in a wide range of temperature (20 to >37 °C). In this study, *G. mellonella* larvae were infected with 5×10^5^ c.f.u. pneumococci grown at different temperatures and the larvae were incubated at 37 °C. The results showed that the mortality rate decreased significantly when the wild-type and the Δ*ciaRcomp* had been cultured at 40 °C (*P*<0.05) compared to bacteria cultured at 34 and 37 °C (Fig. 5). The Δ*ciaR* strain showed a significant attenuation after growth at 34 and 40 °C, relative to 37 °C, and the reduction was more pronounced at 40 °C (*P*<0.05). In addition, Δ*ciaR* was significantly less virulent after growth at 34 and 40 °C than the wild-type (*P*<0.05). The *htrA*-overexpressing wild-type strain (*htrA::htrA-*wt) did not show difference in virulence whatever the growth temperature (*P*>0.05). After growth at 40 °C, the virulence of *htrA::htrA-*wt was significantly higher than that of the wild-type (*P*<0.05). The virulence of Δ*ciaR::htrAcomp* was significantly reduced after growth at 40 °C relative to 34 and 37 °C (*P*<0.01), and the overexpression of *htrA* reconstituted the virulence partially at 40 °C (*P*<0.05).

*In vivo* analysis showed that higher temperature reduces the virulence of pneumococcal strains and the CiaR is required for temperature-independent pneumococcal virulence. Moreover, HtrA can increase the virulence at high temperature.

## Discussion

*Streptococcus pneumoniae* is exposed to different temperatures among different niches of the human host during colonization and invasive disease [3]. Our previous data showed that the pneumococcus responds to thermal fluctuations using a highly complex genetic network [13]. Using microarray analysis, we showed massive alterations in the pneumococcal transcriptome at 34 and 40 °C relative to 37 °C, affecting 252 genes [13]. To further our understanding of pneumococcal thermal adaptation, we focused on the two-component regulatory system CiaR because of its temperature-associated differential expression [upregulated at 34 °C (2.1-fold) and downregulated at 40 °C (2.6-fold), relative to 37 °C]. Specifically, we tested the hypothesis that CiaRH-mediated pneumococcal thermal adaptation occurs through CiaR control over *htrA*.

We highlighted the importance of CiaR and CiaR-regulated *htrA* during thermal adaptation using various pneumococcal traits, including impact on growth, cell size, biofilm formation, as well as production of recognized virulence determinants. Our study showed that the growth of pneumococcal strains was not affected by the temperature changes in a nutrient-rich medium, BHI (Fig. S1), which was not observed in the study by Ibrahim *et al*. [18]. They reported that ∆*ciaR* grew significantly less well than the wild-type strain at 40 °C in BHI, which may be attributed to subtle genetic differences between the D39 strains used in different laboratories. When grown in CDM supplemented with glucose, the growth rate of ∆*ciaR* decreased significantly at 40 °C compared to the wild-type, suggesting that CiaR is required at the higher temperature. One reason for CiaR’s involvement in high-temperature growth can be linked to its control over *htrA*, as overexpression of *htrA* in ∆*ciaR* led to faster growth at 34 and 40 °C than ∆*ciaR* and led to autolysis after the stationary growth phase was reached. This autolytic impact of *htrA* overexpression could be explained by the accumulation of HtrA without CiaR control, and having an inhibitory activity on the cell wall of *S. pneumoniae,* as was previously reported for VvpS, a serine protease in *Vibrio vulnificus* that hydrolases the peptidoglycan residues in the cell wall, leading to autolysis and reduced virulence *in vivo* [36]. However, more work needs to be done to understand whether the accumulation of HtrA and/or the absence of *ciaR* affects cell wall biosynthesis.

CiaRH is involved in biofilm formation in several streptococcal species by affecting the expression of certain genes [37], for example, *speA* (streptococcal pyogenic exotoxin A), in *Streptococcus pyogenes* [38]. In another study, the expression of arginine biosynthesis genes was upregulated in the absence of CiaR, which caused disrupted biofilm in *Streptococcus sanguinis* [39]. However, none of these genes are found to be regulated by CiaR in *S. pneumoniae* (Table S1a), and nor was their expression affected by temperature [13]. One possibility for decreased biofilm formation in the *ciaR* mutant could be related to impaired competence, as the addition of exogenous CSP (competence-stimulating peptide encoded by *comC*) increased the biomass of biofilm during late-exponential growth [40]. In the same study, it was also shown that reduced biofilm formation in ∆*comC* is reconstituted by the addition of exogenous synthetic CSP, suggesting that ComC has a role in pneumococcal biofilm synthesis. As shown in Table S1(a), CiaRH regulates five homologous small non-coding csRNAs (Cia-dependent small RNAs) that enable CiaRH to control the competence by very likely repressing the CSP biosynthesis encoded by *comC* via the expression of *htrA* [41, 42]. It is also noteworthy that competence is repressed through the expression of *htrA* in the absence of csRNAs [42]. Thus, a link between HtrA and csRNAs concerning competence related to biofilm formation in pneumococcus should be studied further.

We observed that the ∆*ciaR* cells were significantly smaller than the wild-type at 34 or 40 °C, showing that CiaR is required for the maintenance of pneumococcal cell size. However, the growth rate of this strain was lower than that of the wild-type. This is contrary to the well-established fact that a reduction in cell size leads to a higher bacterial growth rate due to an increased surface area-to-volume ratio. One possibility for the observed reduced growth rate at different temperatures despite the increased surface area-to-volume ratio could be the adverse impact of temperature on functional metabolic pathways, affecting the cell size of pneumococcus. Protein kinases, which are regulated by TCSs, could also have a role in adjusting cell size, as they generate functional responses by transmitting environmental signals. StkP is a conserved Ser/Thr kinase in pneumococcus and is involved in the localization of cell division apparatus, cell elongation and phosphorylation of cell division protein FtsZ [43], which is mainly regulated by CiaR [43–45]. However, the changes in the transcript level of *stkP* (SPD_1542), *ftsZ* (SPD_1479), or *ftsW* (SPD_0952) were not significant in our previous study [13]. The results also showed that overexpressing *htrA* has no impact on cell size at any temperature. Therefore, it can be concluded that differences observed in cell size occur independently of HtrA, and cell size alterations may occur due to the wider metabolic impact of temperature.

CiaR was found to affect capsule and pneumolysin (Ply) biosynthesis. Pneumolysin is a cholesterol-dependent cytolytic pore-forming toxin expressed during the late log phase of growth [46]. Previous studies showed that haemolysis is temperature-dependent in several species, as specific toxins are differentially activated at different temperatures [47, 48]. Our data showed that haemolytic activity of the wild-type or ∆*ciaR* was significantly reduced at 40 °C compared to 34 °C or 37 °C, and ∆*ciaR* produced less pneumolysin than the wild-type at 40 °C (Fig. 3). Thus, CiaR has an impact on pneumococcal haemolysis at the higher temperature; however, it is not clear how CiaR affects pneumolysin-dependent haemolysis.

In our previous study, we found that the capsule synthesis of the wild-type was higher at 34 °C than at 37 and 40 °C; however, the expression of *cps* locus (SPD_0315 to SPD_0327) was not different at 34 °C relative to 37 °C. We concluded that the inconsistency between the microarray analysis and capsule production is likely related to posttranslational modifications that occur at these temperatures. In this study, when *ciaR* is deleted, the capsule synthesis was significantly diminished at 40 °C. Moreover, ∆*ciaR* produced significantly less capsule at 34 and 40 °C compared to the wild-type (Fig. 4), indicating that the CiaR is involved in the regulation of the capsule indirectly during temperature changes. As shown in Table S1(a), the capsule loci are not targeted by the CiaR. It is possible that the reduction of glucuronic acid at 34 and 40 °C compared to the wild-type was likely due to the indirect effect of mutation on pneumococcal metabolism.

Our *in vivo* study using *G. mellonella* demonstrated that CiaR contributes to the virulence of the pneumococcus at 34 and 40 °C (Fig. 5). This could be due to the impact of temperature on *S. pneumoniae* or the larvae, as it is known that temperature fluctuations affect the production of haemocytes and expression of antimicrobial peptides [49]. The fact that our results showed a reduction of virulence at 34 °C without affecting growth relative to the wild-type might suggest that decreased virulence is due to the deletion of *ciaR* rather than the impact of temperature on larvae (Fig. 1). Our results also revealed that overexpression of *htrA* reconstituted the virulence of ∆*ciaR* partially at 40 °C in larvae.

This study addressed the role of CiaR and CiaR-regulated *htrA* in pneumococcal metabolism and virulence in response to temperature changes. We found that the number of phenotypes was related to the absence of *ciaR* at higher temperature and that HtrA facilitates a degree of reconstitution.

## Supplementary Data

Supplementary material 1
