# Supplementary material for: The involvement of CiaR and the CiaR-regulated serine protease HtrA in thermal adaptation of Streptococcus pneumoniae
Source: Microbiology (Reading). 2023 Feb 22;169(2):001304. doi: 10.1099/mic.0.001304 (PMC10197876; doi:10.1099/mic.0.001304)
Supplement: Supplementary material 1 [file mic-169-1304-s001.pdf]

Supplementary

Table S1a. The summary of CiaR-regulated genes and operons (Halfmann et al., 2007; Mascher et al., 2003; Slager et al., 2019).

Gene annotation number	Gene	Product
SPD_0026	*ccnC*	Small non-coding csRNA3
	ccnE	Small non-coding csRNA5
	ccnA	Small non-coding csRNA1
	ccnB	Small non-coding csRNA2
	ccnD	Small non-coding csRNA4
SPD_0098		Glycosyltransferase, group 2 family
SPD_0138		Glycosyltransferase, group 1 family
SPD_0222-0223		Phosphoglycerate mutase family protein
		Iron(III) ABC transporter, permease protein
SPD_0241	*ruvB*	Holliday junction DNA helicase
SPD_0262-0264	*manN*	PTS system, mannose/fructose/sorbose family protein, IID component
	manM	PTS system, mannose-specific IIC component
	manL	PTS system, mannose-specific IIAB components
SPD_0466-0471		Hypothetical protein
	blpS	BlpS protein
	blpR	Response regulator BlpR
	blpH	Histidine kinase BlpH
	blpC	Peptide pheromone BlpC
	blpB	Pseudogene
SPD_0478-0483	*rimP*	Ribosome maturation factor RimP
	nusA	Transcription termination factor NusA
		Hypothetical protein
		Putative transcription termination protein
	infB	Ribosomal protein L7A family protein
	rbfA	Ribosome-binding factor A
SPD_0532-0537	*RecJ*	Single-stranded-DNA-specific exonuclease
		Metallo-beta-lactamase superfamily protein
	estA	Tributyrin esterase
	murM	Merine/alanine-adding enzyme
	_fibA	Beta-lactam resistance factor
	fibB	Matrixin family protein
SPD_0701-0702	*ciaR*	DNA-binding response regulator CiaR
	ciaH	Sensor histidine kinase CiaH
SPD_0775		Hypothetical protein
SPD_0733-0739	*coaA*	Pantothenate kinase
		Membrane protein
		Methyltransferase small domain
	pdp	Pyrimidine-nucleoside phosphorylase

	deoC<br>*cdd-1*	Deoxyribose-phosphate aldolase<br>Cytidine deaminase<br>Putative membrane lipoprotein TmpC precursor
SPD_0868		Protease maturation protein, putative
SPD_0913-0920	 *rumA*	Hypothetical protein<br>23S rRNA (uracil-5-)-methyltransferase RumA<br>Iron-compound ABC transporter, binding protein<br>Iron-compound ABC transporter, permease protein<br>Iron-compound ABC transporter, ATP-binding protein<br>Hypothetical proteins (_0919/0920)
SPD_0941-0951		Hypothetical proteins<br>AMP-binding enzyme, putative (_0945)
SPD_1122-1131	*dprA*<br>*licC*<br>*licB*<br>*licA/pck*<br>*tarJb/ispD*<br>*tarIb*<br><br><br>*licD1*<br>*licD2*<br>*carB*	DNA processing protein DprA, putative<br>CTP:phosphocholine cytidylyltransferase<br>Protein LicB<br>Choline kinase<br>Ribulose-5-phosphate reductase<br>2-C-methyl-D-erythritol 4-phosphate cytidylyltransferase<br>Polysaccharide biosynthesis protein, putative<br>Phosphotransferase LicD1<br>Phosphotransferase LicD2<br>Carbamoyl-phosphate synthase, large subunit
SPD_1324-1327	 <br>*pgm*<br><br>*bta*	IS630-Spn1, transposase Orf2<br>Pseudogene<br>Phosphoglucomutase/phosphomannomutase family protein<br>Bacterocin transport accessory protein
SPD_1505-1507	 *axe-1*<br>*recG*	Hypothetical protein<br>Acetyl xylan esterase, putative<br>ATP-dependent DNA helicase RecG
Downstream of SPD_1582		Lipoproteins
SPD_1744-1753	 <br><br><br>*wrbA*	Lipoprotein<br>Transcriptional regulator PlcR, putative<br>Hypothetical proteins (_1746/1747/1748)<br>Bacteriocin formation protein, putative<br>Multimeric flavodoxin WrbA<br>Membrane protein, putative<br>Toxin secretion ABC transporter, ATP-binding/permease protein<br>Serine protease, subtilase family protein
SPD_1769		Membrane protein, putative
SPD_1799-1802	*desK*	Sensor histidine kinase, putative<br>Membrane protein, putative<br>ABC transporter, ATP-binding protein<br>Hypothetical protein
SPD_1930-1934	 *malP*<br>*malQ*	Hypothetical proteins (_1930/1931)<br>Maltodextrin phosphorylase<br>4-alpha-glucanotransferase

	malX	Maltose/maltodextrin ABC transporter, maltose/maltodextrin-binding protein
SPD_2002-2006	*dltD*<br><br>*dltC*<br>*dltB*<br>*dltA*<br>*dltX*	Undecaprenol-phosphate-poly(glycerophosphate subunit) D-alanine transfer protein<br>D-alanine-poly(phosphoribitol) ligase subunit 2<br>Protein DltB<br>D-alanine--poly(phosphoribitol) ligase subunit 1<br>D-Alanyl-lipoteichoic acid biosynthesis protein
SPD_2060-2069	 <br><br>*comE*<br>*comD*<br>*comC1*<br><br><br>*htrA*<br>*spoJ*	Transcriptional regulator, TetR family protein<br>tRNA-Asn<br>tRNA-Glu<br>Response regulator<br>Putative sensor histidine kinase ComD<br>Competence-stimulating peptide type 1<br>tRNA-Arg<br>Hypothetical protein<br>Serine protease<br>ParB family transcriptional regulator, chromosome partitioning protein
SPD_2378	*srf-1*	ncRNA of unknown function
	prsA	Putative parvulin type peptidyl-prolyl isomerase*

* Downstream of ComX-binding element, *S. pneumoniae* INV200 (GenBank accession number FQ312029.1) (Slager et al., 2019).

Table S1b. Fold difference of CiaR-regulated genes at 34°C and 40°C relative to 37°C shown in our previous study (Gazioglu et al., 2021). Blue represents upregulation and red represents downregulation of the genes.

Gene annotation number	Gene	Product	34°C	40°C
SPD_0466		Hypothetical protein	7.01	-6.49
SPD_0467	*blpS*	BlpS protein	3.21	-2.84
SPD_0468	*blpR*	Response regulator BlpR	2.49	-2.41
SPD_0470	*blpC*	Peptide pheromone BlpC	3.38	-2.02
SPD_0701	*ciaR*	DNA-binding response regulator CiaR	2.06	-2.59
SPD_0702	*ciaH*	Sensor histidine kinase CiaH	1.96	-2.24
SPD_0775		Hypothetical protein	2.35	
SPD_0913		Hypothetical protein	3.21	-2.75
SPD_1122	*dprA*	DNA processing protein DprA, putative	8.13	-6.99
SPD_1131	*carB*	Carbamoyl-phosphate synthase, large subunit	1.9	
SPD_1744		Lipoprotein	3.1	-3.22
SPD_2006	*dltX*	D-Alanyl-lipoteichoic acid biosynthesis protein	2.52	
SPD_2063	*comE*	Response regulator	39.75	-25.36

SPD_2064	*comD*	Putative sensor histidine kinase ComD	53.65	-44.92
SPD_2065	*comC1*	Competence-stimulating peptide type	35.18	-23.95
SPD_2068	*htrA*	Serine protease	6.38	-3.73
SPD_2069	*spoJ*	ParB family transcriptional regulator, chromosome partitioning protein	4.4	-3.5

Table S2. List of strains and plasmids used in this study.

Strain	Description	Source
D39 (WT)	Serotype 2 strain	NCTC 7466
Δ*ciaR*	D39; SPD_0701:Spec^R	this study
Δ*ciaRcomp*	Intact copy of *ciaR* + Δ*ciaR*::Spec^R; Kan^R	this study
htrA::htrA-wt	Intact copy of *htrA* + D39::Spec^R; Kan^R	this study
Δ*ciaR::htrAcomp*	Intact copy of *htrA* + Δ*ciaR*::Spec^R; Kan^R	this study
E. coli DH5α	Contains antibiotic resistance genes; plasmid propagation	Novagen,UK
E.coli BL21 DE3	*F-ompT hsdsB (rb- mb-) gal dcm (DE3)*	Novagen,UK
**Plasmids**	**Description**	**Source**
pCEP	Genetic complementation; Kan^R	Guiral et al., 2006
pDL278	Amplification of Spec^R (*aadA*)	Yesilkaya et al., 2000

Table S3. Oligonucleotide primers used in this study. Bold typeface shows homologues recombination sites of antibiotic cassette. Bold and underlined typeface show incorporated restriction sites.

Primers	Sequence
spec/F<br>spec/R	ATCGATTTTCGTTCGTGAAT<br>GTTATGCAAGGGTTTATTGT
LF/F_*ciaR*<br>LF/R_*ciaR*	AGAGTCTTATCTGGTGGTTTCAGCT<br>**TATTCACGAACGAAAATCGAT**CTCTCTGCATTTTACATGAGATAGC
RF/F_*ciaR*<br>RF/R_*ciaR*	**AACAATAAACCCTTGCAT**GTTCAGTAAACTTAAAAAA<br>ACATGGAGCTCCGGCTTAATCCCATCATCTC
F_Comp2068<br>R_Comp2068	CG<u>**CCATGG**</u>CAATTAAGAAAATCACAA<br>CG<u>**GGATCC**</u>TTAAATTCTAAATCACCT
Mal/F<br>pCEP/R	GCTTGAAAAGGAGTATACTT<br>AGGAGACATTCCTTCCGTATC

Figure S1. Growth profiles of pneumococcal strains in BHI at 34°C, 37°C, and 40°C. Error bars show the standard error of the mean for three individual measurements. No significant differences in growth profiles of the strains at both temperatures were recorded.

34°C

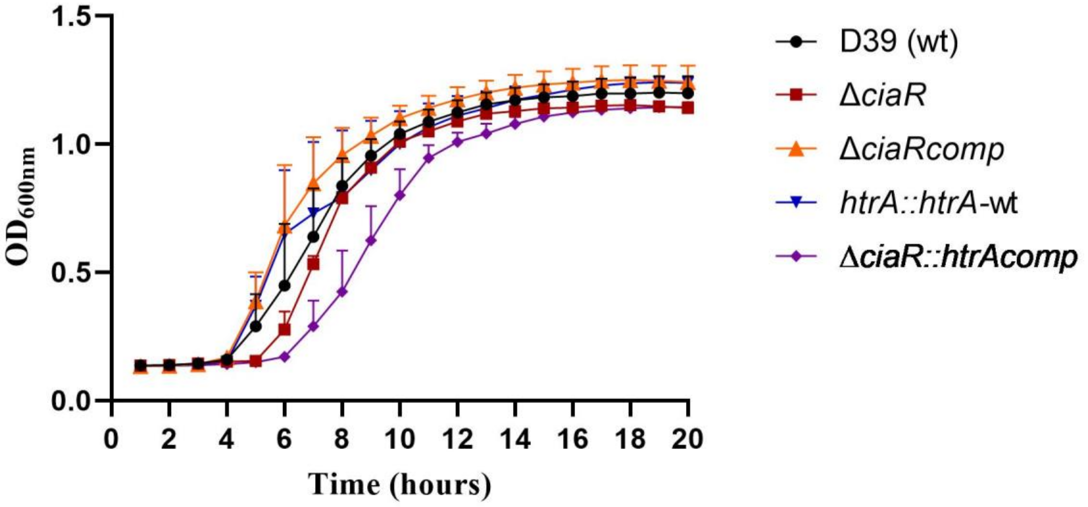

37°C

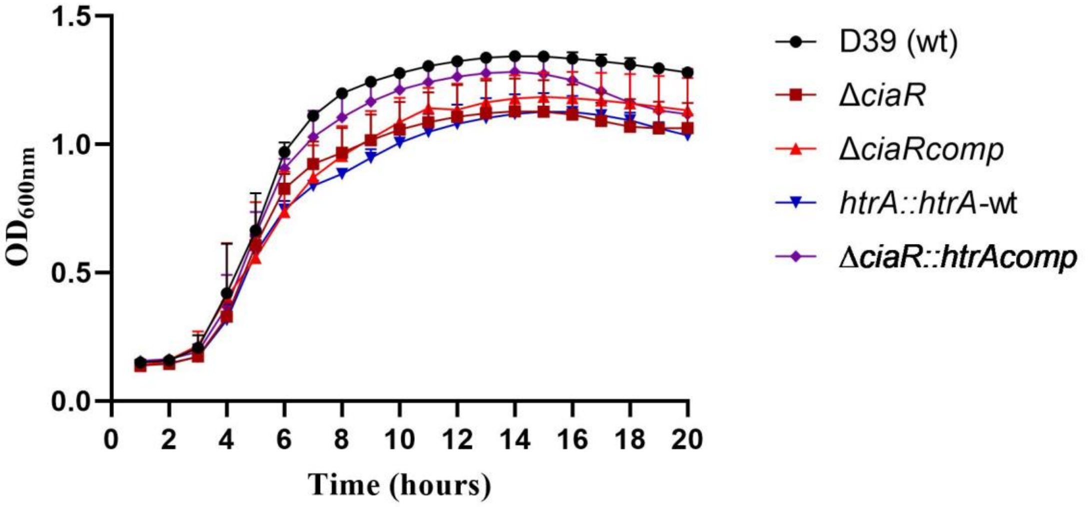

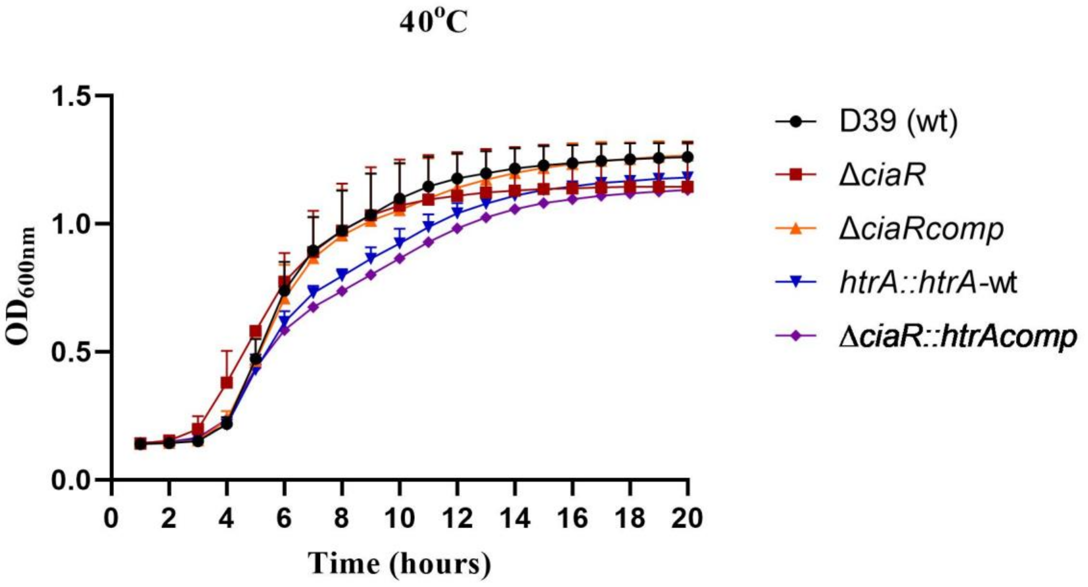
40°C
OD600nm
Time (hours)
D39 (wt)
ΔciaR
ΔciaRcomp
htrA::htrA-wt
ΔciaR::htrAcomp
0.0
0.5
1.0
1.5
0 2 4 6 8 10 12 14 16 18 20
